# GPNMB is expressed in human epidermal keratinocytes but disappears in the vitiligo lesional skin

**DOI:** 10.1038/s41598-020-61931-1

**Published:** 2020-03-18

**Authors:** Kazal Boron Biswas, Aya Takahashi, Yukiko Mizutani, Satoru Takayama, Asako Ishitsuka, Lingli Yang, Fei Yang, Arunasiri Iddamalgoda, Ichiro Katayama, Shintaro Inoue

**Affiliations:** 1 Department of Cosmetic Health Science, Gifu Pharmaceutical University, Gifu, Japan; 2 Department of Research and Development, Ichimaru Pharcos Co. Ltd., Motosu Gifu, Japan; 3 Department of Dermatology, Osaka University School of Medicine, Osaka, Japan

**Keywords:** Vitiligo, Cell adhesion, Glycoproteins

## Abstract

GPNMB is involved in multiple cellular functions including cell adhesion, stress protection and stem cell maintenance. In skin, melanocyte-GPNMB is suggested to mediate pigmentation through melanosome formation, but details of keratinocyte-GPNMB have yet to be well understood. We confirmed the expression of GPNMB in normal human epidermal keratinocytes (NHEKs) by reducing the expression using siRNA. A higher calcium concentration of over 1.25 mM decreased the GPNMB expression. Histological staining showed that GPNMB was expressed in the basal layer of normal skins but completely absent in vitiligo skins. The normal expression of GPNMB in nevus depigmentosus skin suggested that lack of GPNMB is characteristic of vitiligo lesional skins. IFN-γ and IL-17A, two cytokines with possible causal roles in vitiligo development, inhibited GPNMB expression *in vitro*. Approximately 4–8% of the total GPNMB expressed on NHEKs were released possibly by ADAM 10 as a soluble form, but the process of release was not affected by the cytokines. The suppressive effect of IFN-γ on GPNMB was partially via IFN-γ/JAK2/STAT1 signaling axis. Decreased GPNMB expression in keratinocytes may affect melanocyte maintenance or survival against oxidative stress although further studies are needed. These findings indicate a new target for vitiligo treatment, focusing on the novel role of IFN-γ and IL-17 in downregulating keratinocyte-GPNMB.

## Introduction

GPNMB (Glycoprotein nonmetastatic melanoma protein B) – also known as osteoactivin, dendritic cell-heparin integrin ligand (DC-HIL), or hematopoietic growth factor inducible neurokinin-1 type – is a type I transmembrane glycoprotein. GPNMB has 2 transcript variants encoding 560 and 572 amino acid isoforms in human and shares a 25% amino acid sequence homology with PMEL-17, a melanocyte-specific melanosomal protein^1^. The extracellular part of GPNMB mainly contains an RGD motif that binds to integrin in the process of maintaining cell-cell adhesion, and an Ig-like polycystic kidney disease (PKD) domain involved in protein-protein and protein-carbohydrate interactions. The cytoplasmic tail contains an immunoreceptor tyrosine-based activation motif (ITAM) that takes part in the intracellular signaling via Src and Syk cytoplasmic kinases, and a di-leucine motif required for its endosomal⁄melanosomal sorting signal^2^.

GPNMB is widely expressed in various tissues such as the skin, brain, breasts, muscle, and bone^3–7^. The known functions of GPNMB include cellular adhesion through integrin^1^, regulation of the degeneration/regeneration of the extracellular matrix in skeletal muscles^6^, the mineralization of bone^2^, the differentiation of osteoclasts^8^ and osteoblasts^9^, the impairment of T-cell activation^10^, the regulation of inflammatory responses in macrophages^11^, the suppression of motor neuron degeneration in amyotrophic lateral sclerosis^12^, and the invasion and metastasis of several cancers^13–17^. GPNMB has also been widely demonstrated to increase the endoplasmic reticulum (ER) stress response by inducing the expression of glucose regulated protein (GRP78/BiP) in the brain^18^.

The extracellular fragments of GPNMB are known to be cleaved by a disintegrin and metalloproteases 10 (ADAM10) on the plasma membrane in the process of ectodomain shedding and secreted into the extracellular spaces^19^. This shed GPNMB then mediates signal transduction via cell surface proteins such as Na^+^/K^+^-ATPase^20^ and CD44^21^ as receptors for GPNMB. The extracellular fragment of GPNMB shows neuroprotective effects by activating the phosphoinositide 3-kinase (PI3K)/Akt and mitogen-activated protein kinase (MAPK)-extracellular signal-regulated kinase (ERK) kinase (MEK)/ERK pathways via binding to α-subunit of Na^+^/K^+^-ATPase in the neuronal cell line^20^. According to one recent report, the soluble form of GPNMB derived from B16 melanoma cells travels to the distal organs and promotes the metastatic capacity of tumor cells by excluding T-lymphocytes from the pre-metastatic niches^22^. Moreover, the extracellular part of GPNMB has shown a neuroprotective property by attenuating astrocyte-mediated neuroinflammation in a CD44-dependent manner in mouse^21^.

In skin, GPNMB is predominantly expressed in the melanocytes and regulated by microphthalmia-associated transcription factor (MITF)^23^. Melanocyte-GPNMB is present in all stages (I–IV) of melanosomes^24^, and the silencing of its expression by siRNA inhibits the formation of melanosomes^25^, indicating its critical role in pigmentation. GPNMB also functions as an adhesion protein between melanocytes and keratinocytes through integrin^1^.

The foregoing studies raise the intriguing possibility that GPNMB plays a role in depigmentation disorders. A loss of GPNMB has been shown to cause autosomal-recessive amyloidosis cutis dyschromica, which is mainly characterized by hyperpigmentation mottled with hypopigmented macules in human skin^26^. In vitiligo, the melanocytes detached from the basal layer and moved to the suprabasal layer of the epidermis in response to a decrease in the levels of melanocyte E-cadherin, an adherent molecule with keratinocytes^27^.

Although GPNMB is known as a melanosome-specific, melanocytic cell marker protein^19^, its expression and function in skin keratinocytes is still controversial. Tomihari *et al*. detected the expression of GPNMB in skin keratinocytes by immunostaining skin biopsy samples from healthy human adult^1^. Immunostaining data by another group, however, have suggested that GPNMB is exclusively expressed in skin melanocytes, and absent in both skin keratinocytes and fibroblasts^19^.

In the first part of the present study we confirmed an antibody that showed specific signals that were abolished by GPNMB-siRNA treatment. We then clarified the expression of GPNMB in normal human epidermal keratinocytes in culture and showed that ADAM10 was responsible for the shedding of GPNMB in the medium. We also showed that GPNMB was expressed in healthy skin epidermis and in nevus depigmentosus skin, but it was absent in the lesional epidermis of vitiligo patients. Moreover, we provided evidences that IFN-γ and IL-17A, two cytokines with possible causal roles in vitiligo development, inhibited GPNMB expression *in vitro*. Therefore, this report on keratinocyte-GPNMB may provide new insights into pathophysiology of depigmented disorders like vitiligo for clinical applications.

## Results

### Characterization of GPNMB antibody specificity

We began our study by characterizing the antibody for western blot (WB) analysis and cell immunostaining of GPNMB. When several melanoma cells (C32TG, G361, and Mewo) were used as GPNMB-positive control cells, WB analyses in both the presence and absence of glycosidase (PNGase) were consistent with previous reports^1,19,28^, showing similar patterns of bands corresponding to precursor and mature forms of glycosylated GPNMB, as well as deglycosylated forms (Supplementary Fig. S1a). For normal human epidermal melanocytes (NHEMs), the specificity of the same antibody was proved by small-interfering RNA (siRNA), which decreased GPNMB mRNA significantly (p < 0.01) (Supplementary Fig. S1b) with concomitant decreases of the signal corresponding to GPNMB in WB (Supplementary Fig. S1c). Moreover, immunostaining of NHEMs using the same primary antibody showed positive GPNMB signal (Supplementary Fig. S1d). Altogether these data proved that the antibody for GPNMB used in this study was specific, and might therefore be working properly.

### Identification of GPNMB expression in normal human epidermal keratinocytes (NHEKs)

**Figure 1:**
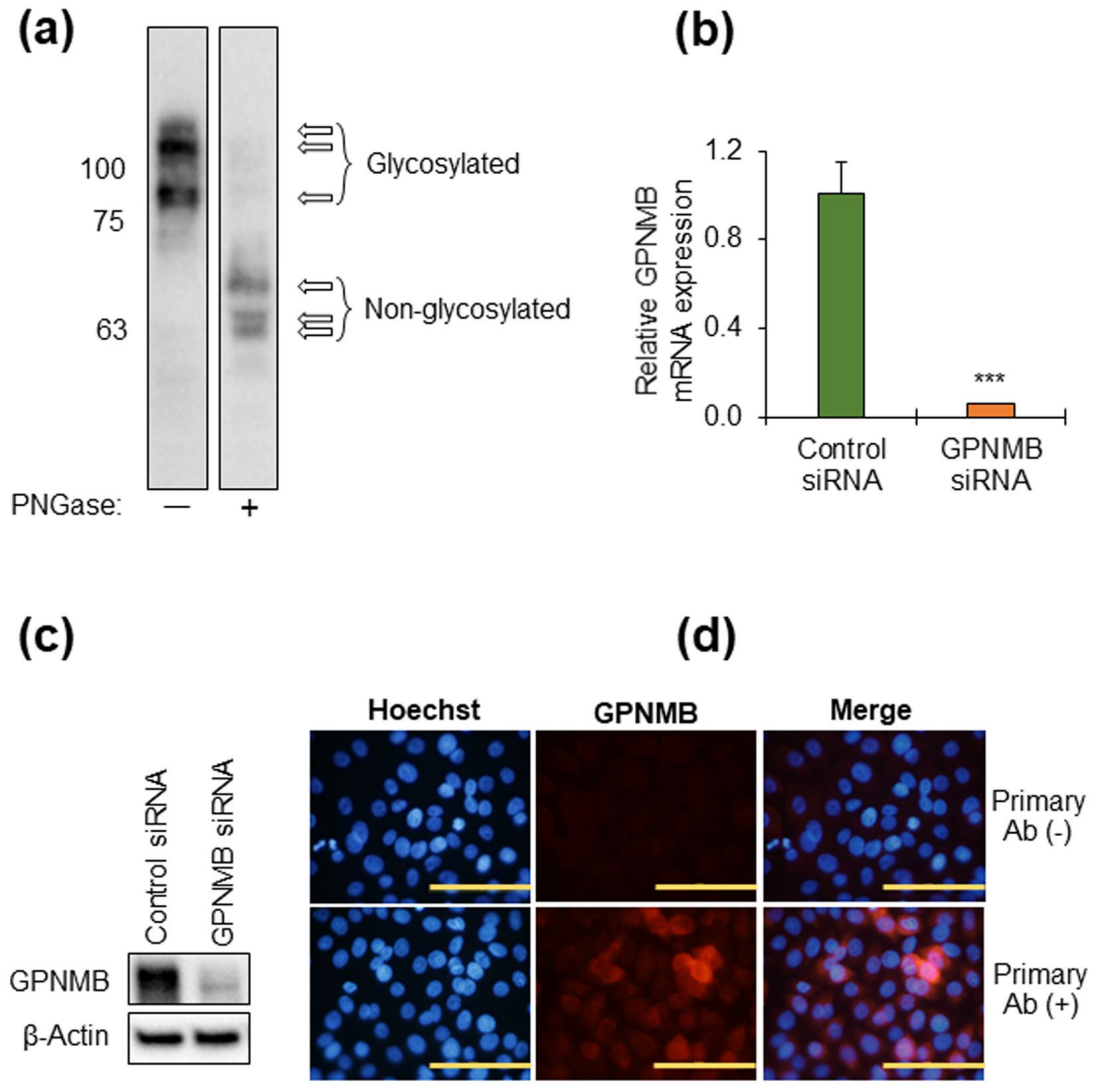
GPNMB is expressed in normal human epidermal keratinocytes (NHEKs). (**a**) NHEKs were lysed and total protein was isolated from the cell lysate. Glycosylated (PNGase-untreated) and non-glycosylated (PNGase-treated) GPNMB was identified by western blotting. The two lanes were cropped from two different parts of the same blot. The full-length blot has been presented in Supplementary Fig. S8. (**b**) NHEKs were transfected with control-siRNA and GPNMB-siRNA for 48 hrs. The mRNA expression of GPNMB was measured by real-time PCR. Data were expressed as mean ± SD (n = 3). ***P < 0.001 vs control (Student’s t-test). (**c**) Under the same experimental conditions described in (**b**), total protein was isolated from the cell lysate, treated with PNGase, and subjected to WB analyses. The full-length blot has been presented in Supplementary Fig. S9. (**d**) NHEKs were cultured on glass-bottom dishes and immunostained for GPNMB expression in the presence or absence of GPNMB primary antibody. The scale bar is equal to 100 µm.

The GPNMB antibody which was proved specific in melanoma and melanocyte cells (as positive controls) was used for detecting WB band and immunostaining signal in NHEKs. WB data showed three bands of GPNMB in the absence and presence of PNGase corresponding to glycosylated and deglycosylated forms of the GPNMB, respectively (Fig. 1a, and Supplementary Fig. S8). Moreover, GPNMB siRNA, which depleted mRNA significantly (p < 0.001) (Fig. 1b), decreased substantially the GPNMB signal in WB (Fig. 1c, and Supplementary Fig. S9). Furthermore, immunostaining of NHEKs also showed positive GPNMB signal (Fig. 1d). These results indicate that cultured NHEKs express GPNMB in both mRNA and protein levels.

### Effect of Ca^2+^ on the expression of GPNMB in NHEK

**Figure 2:**
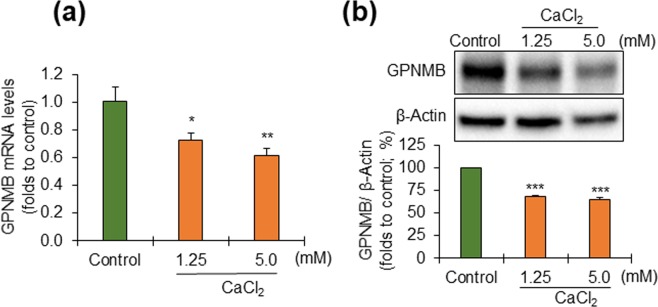
Proliferative NHEKs express more GPNMB than differentiated NHEKs. NHEKs were cultured in the presence of high concentrations of calcium for 24 hrs. (**a**) The dose-dependent effect of calcium on the expression of GPNMB mRNA was measured by real-time PCR. The data were expressed as mean ± SD (n = 3). *P < 0.05 and **P < 0.01 vs control (Student’s t-test). (**b**) Total protein was extracted from the cell lysates, treated with PNGase, and subjected to western blot analysis. The protein levels of GPNMB were quantified relative to those of β-actin. The data were expressed as mean ± SD (n = 3). ***P < 0.001 vs control (Student’s t-test). The full-length blot has been presented in Supplementary Fig. S10.

To explore whether differentiated or non-differentiated NHEKs express GPNMB, we investigated the effects of different concentrations of Ca^2+^ (0.06, 1.25, and 5.0 mM) on the expression of GPNMB. The expression of GPNMB at both the mRNA (Fig. 2a) and protein (Fig. 2b, and Supplementary Fig. S10) levels was decreased at higher Ca^2+^ concentration (1.25 and 5.0 mM) compared with the lower Ca^2+^ concentration condition (0.06 mM), indicating that non-differentiated keratinocytes express GPNMB at higher level than differentiated cells.

### GPNMB expression in healthy and depigmented epidermis

**Figure 3:**
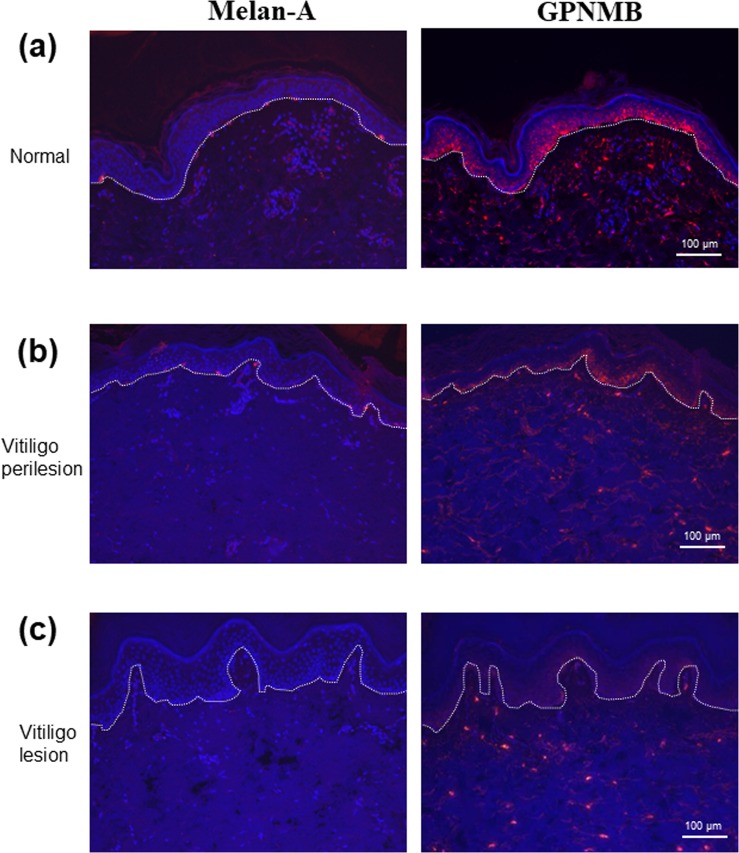
GPNMB is expressed in normal skin, but disappears in vitiligo skin. Skin samples collected from normal skin, vitiligo perilesional skin, and vitiligo lesional skin were immunostained using anti-human GPNMB antibody. The dotted white line depicts the location of the dermal-epidermal junction. GPNMB was stained red, Melan-A stained red, and DAPI was stained blue.

Next, we investigated the expression of GPNMB in the epidermis of healthy skins and in depigmented skins from vitiligo and nevus depigmentosus patients. GPNMB was expressed in keratinocytes in the basal layer of healthy human skin epidermis, although the staining intensity was weaker than that of melanocytes (Fig. 3a). Intriguingly, GPNMB signals were abolished in the basal layer keratinocytes of vitiligo lesions, whereas the signals in prelesional regions were maintained at the same levels as those in healthy skin (Fig. 3b,c). Similar results were also shown in Supplementary Fig. S3. On the contrary, GPNMB signals remained positive in the lesional epidermis of nevus depigmentosus skins (Supplementary Fig. S3), indicating that the loss of epidermal GPNMB was unique to vitiligo depigmentation.

### Production of soluble GPNMB and the effects of Ca^2+^ and ADAM inhibitors

**Figure 4:**
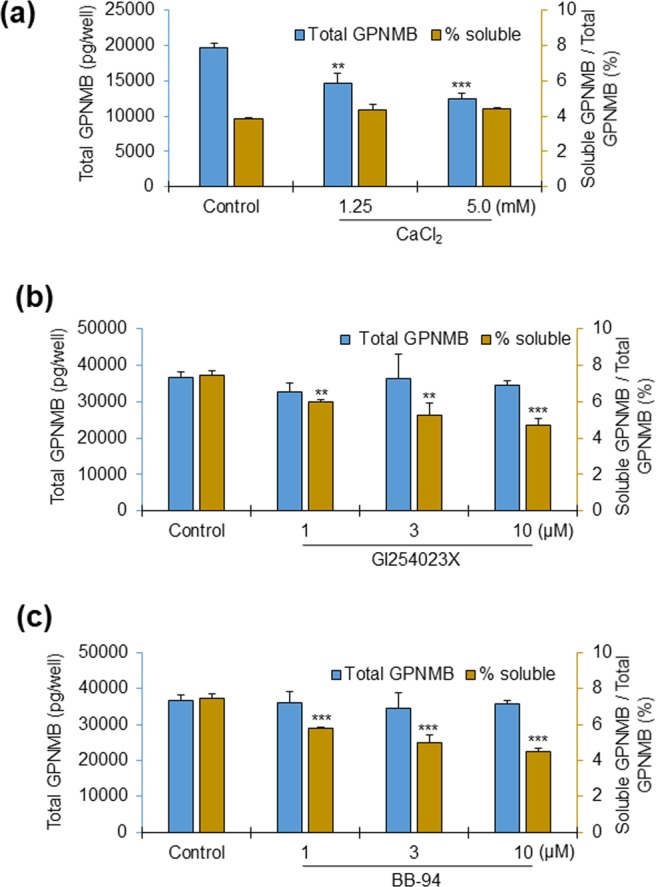
GPNMB is released in the cultured medium of NHEKs, and the release is regulated by ADAMs. Cultured medium was collected (for sGPNMB), and cell lysate protein was prepared from the corresponding wells (for cell-associated GPNMB). The absolute concentrations of the soluble and cell-associated GPNMBs were measured by ELISA. The data were presented as the total GPNMB (sGPNMB + cell-associated GPNMB) and the % release of sGPNMB compared with the total GPNMB. (**a**) 24 hrs after the incubation of NHEKs with different concentrations of CaCl_2,_ the soluble and total GPNMB were measured. Data were expressed as mean ± SD (n = 3). *P < 0.05, **P < 0.01, and ***P < 0.001 vs control (Student’s t-test). (**b**) Soluble and cell-associated GPNMB was measured by ELISA after the NHEKs were incubated in the presence of different concentrations (1, 3, and 10 µM) of GI254023X (an ADAM10 inhibitor). Data were presented as soluble GPNMB and the % ratio of soluble to total GPNMB, and expressed as mean ± SD (n = 3). **P < 0.01 and ***P < 0.001 vs control (one-way ANOVA followed by Dunnett’s test). (**c**) The dose-dependent effect of BB-94 (a broad spectrum ADAM inhibitor) on the shedding of sGPNMB was determined by measuring the concentrations of GPNMB in the cultured medium and the cell lysate by ELISA. Data were expressed as mean ± SD (n = 3). **P < 0.01 and ***P < 0.001 vs control (one-way ANOVA followed by Dunnett’s test).

To account for the possibility that the loss of GPNMB signals in vitiligo epidermal keratinocytes stems from enzymatic cleavage or the secretion of cell-associated GPNMB, we examined the capacity of keratinocytes to release soluble GPNMB (sGPNMB). First, we measured the amount of sGPNMB in the cultured medium of NHEKs, as well as the amount of cell-associated GPNMB by enzyme-linked immunosorbent assay (ELISA) in the presence of low and high Ca^2+^ concentrations. NHEKs released about 4–8% of the total GPNMB in the medium when they were exposed to both low and high Ca^2+^ concentrations, although the total GPNMB (the sum of soluble and cell-associated GPNMB) was decreased in the high-calcium medium (Fig. 4a). The Ca^2+^-dependent decrease of cell-associated GPNMB determined by ELISA correlated well with the decreases shown by WB (Fig. 2b). These data suggest that proliferative NHEKs express higher GPNMB compared to differentiated NHEKs, whereas the ratio of sGPNMB production keeps constant.

To clarify whether sGPNMB is produced proteolytically, we examined the effects of ADAM inhibitors on the amount of sGPNMB. We used two ADAM inhibitors – one is a selective inhibitor for ADAM10 (namely GI254023X), and another is a broad spectrum inhibitor for ADAMs and matrix metalloproteases (namely BB-94). Both inhibitors inhibited the sGPNMB release dose-dependently without affecting the amount of total GPNMB (Fig. 4b,c). The inhibitory effect was partial but significant.

This result suggests that some of the membrane-bound GPNMBs of NHEKs are cleaved by ADAM10 to produce sGPNMB. If they are, the process is consistent with the previously reported finding that GPNMB is cleaved by ADAM10 and secreted extracellularly in breast cancer cells^29^.

### Effects of IFN-γ on GPNMB expression and sGPNMB production in NHEKs

**Figure 5:**
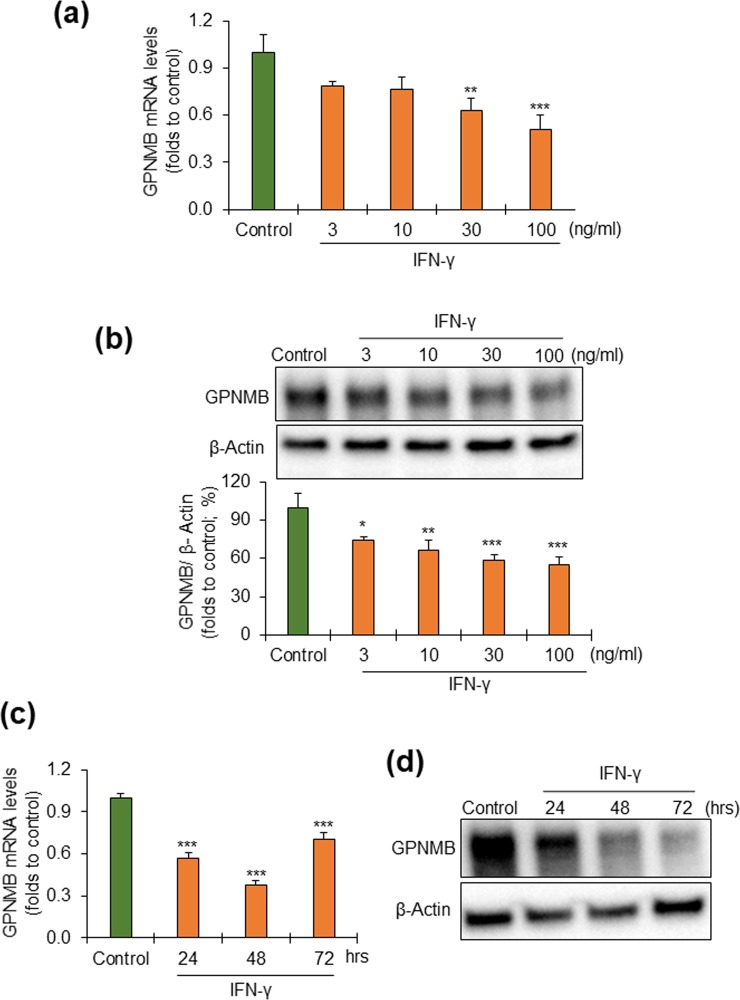
The expression of GPNMB in NHEKs is downregulated by IFN-γ in a concentration- and time-dependent manner. (**a,b**) To observe the dose dependency, the cells were incubated with different concentrations (0, 3, 10, 30, and 100 ng/ml) of IFN-γ for 24 hrs. Next, (**a**) the mRNA expression of GPNMB was determined by real-time PCR, and **(b)** cell-associated GPNMB, treated with PNGase, was analyzed by western blotting followed by quantification of the protein relative to β-actin. The full-length blot has been presented in Supplementary Fig. S11. Data were expressed as mean ± SD (n = 3). *P < 0.05, **P < 0.01, and ***P < 0.001 vs control (one-way ANOVA followed by the Tukey’s test). **(c**,**d)** The time-dependent effect of IFN-γ was observed by incubating the cells with 30 ng/ml of IFN-γ for different periods (0, 24, 48, and 72 hrs). **(c)** The mRNA expression of GPNMB was determined by real time PCR, and **(d)** cell-associated GPNMB, treated with PNGase, was analyzed by western blotting. The full-length blot has been presented in Supplementary Fig. S12. Data were expressed as mean ± SD (n = 3). ***P < 0.001 vs control (one-way ANOVA followed by Tukey’s test).

In our next experiments we sought to explain the disappearance of GPNMB signals in vitiligo lesional epidermis by examining how several cytokines and chemokines potentially involved in vitiligo pathophysiology affected GPNMB expression and sGPNMB production in NHEKs. Among the factors tested, only IFN-γ was shown to decrease the GPNMB expression dose-dependently at both mRNA (Fig. 5a) and protein levels (Fig. 5b, and Supplementary Fig. S11). The effects were partial but significant, and time-dependent (Fig. 5c,d, and Supplementary Fig. S12). The ELISA determination of sGPNMB, however, showed that IFN-γ had no effect on the release of sGPNMB into the cultured medium of NHEKs (Supplementary Fig. S4a).

Among the other cytokines, only IL-17A downregulated the expression of GPNMB (Supplementary Fig. S4b), while the others (IL-1β, IL-6, and TNF-α) left the expression of GPNMB unchanged (Supplementary Fig. S5). The chemokines CXCL10, CXCL12, and CXCL16 likewise showed no effects on GPNMB expression under the same experimental conditions (Supplementary Fig. S6).

### Receptor involvement in the action of IFN-γ on the suppression of GPNMB expression

**Figure 6:**
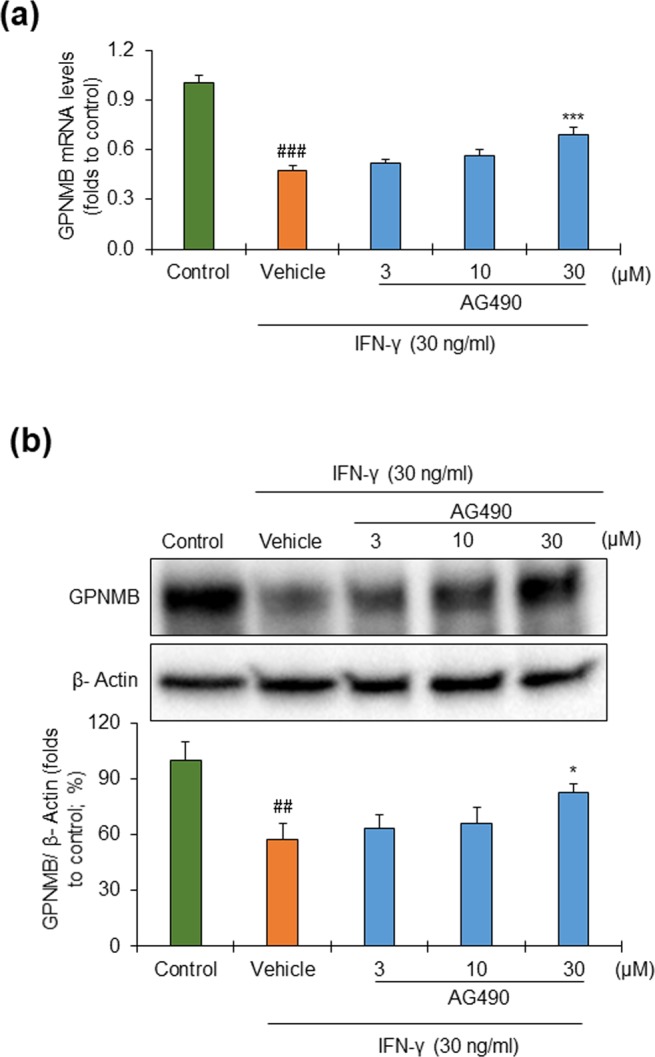
IFN-γ acts through the JAK2/STAT1 signaling pathway to suppress GPNMB expression. NHEKs were incubated with different concentrations (3, 10, and 30 µM) of AG490 (JAK2 inhibitor) for 6 hrs and then incubated with 30 ng/ml of IFN-γ for 24 hrs. The control cells were left untreated, but the vehicle contained IFN-γ (30 ng/ml). (**a**) The mRNA expression of GPNMB was determined by real-time PCR, and **(b)** cell-associated GPNMB (non-glycosylated) was analyzed by western blotting followed by quantification of the protein relative to β-actin. The full-length blot has been presented in Supplementary Fig. S13. Data were expressed as mean ± SD (n = 3) and analyzed by one-way ANOVA followed by the Tukey’s HSD test. ^##^*P* < 0.01 and ^###^*P* < 0.001 vs control; *P < 0.05 and ***P < 0.001 vs vehicle.

To confirm whether the action of IFN-γ on GPNMB expression is receptor dependent, we examined the effects of AG490, a potent Janus Activated Kinase (JAK) 2 inhibitor. AG490 restored the IFN-γ-induced downregulation of GPNMB dose dependently at both the mRNA (Fig. 6a) and protein (Fig. 6b, and Supplementary Fig. S13) levels. Moreover, suppression of STAT1 by siRNA transfection showed a clear tendency of inhibiting IFN-γ effect on the down-regulation of GPNMB expression (Supplementary Fig. S7). These data suggest that the JAK2/STAT1 signaling pathway may be involved in regulating the expression of GPNMB. However, there must be other pathways involved as well, as the AG490 treatment increased the levels of GPNMB against IFN-γ alone, but did not reach levels as in control cells.

## Discussion

We began our study by verifying and validating the specificity of anti-GPNMB antibody in our own experimental systems using melanoma cells, as well as normal human epidermal melanocytes (NHEMs), a cell type in which GPNMB expression has been well characterized^1,19,28^, as positive controls. The specificity of this antibody was finally confirmed by the disappearance of signals corresponding to GPNMB in WB and cell immunostaining analyses when GPNMB mRNA was knocked down by the specific siRNA in NHEMs (Supplementary Fig. S1).

We then proved, through observation, that the expression of GPNMB in NHEKs using the validated and specific anti-GPNMB antibody (Fig. 1) was the same as that of melanoma cells and NHEMs (Supplementary Fig. S1). This result agrees with the report by Tomihari *et al*.^1^, but the controversial finding by another group that epidermal keratinocytes lacked GPNMB expression in immunostained skin^19^ might have stemmed from the difference of the antibody used. The higher expression of GPNMB found in cultured NHEKs at the lower Ca^2+^ concentration (Fig. 2) explains the normal expression of GPNMB in proliferative keratinocytes and is consistent with the strong signal strength in the basal epidermis of human skins (Fig. 3a).

We found that keratinocytes in the basal layer of the vitiligo lesion lost the GPNMB signals although perilesional keratinocytes retained the positive signals (Fig. 3b), suggesting that GPNMB disappearance from keratinocytes is closely correlated with the loss of melanocytes. However, the lesional epidermis of nevus depigmentosus skins showed positive signals in spite of the loss of melanocytes (Fig. 3c), indicating that the disappearance of epidermal GPNMB is specific to vitiligo depigmentation.

These findings imply that vitiligo-related factors may be involved in the downregulation of keratinocyte GPNMB. We found that the expression of GPNMB was downregulated by IFN-γ, as well as IL-17A. While our findings show that the actions of IFN-γ were mediated at least through the IFNGR signaling pathway via JAK2/STAT1, further investigation will be needed to identify a downstream target of the JAK2/STAT1 signaling pathway to suppress GPNMB mRNA expression. IFN-γ is one of the candidate cytokines involved in vitiligo pathology. IFN-γ signaling has been reported to mediate hypopigmentation of primary human melanocytes by arresting melanosome maturation^30^. Tulic *et al*. recently reported that type-1 innate lymphoid cells (NK and ILC1) were elevated in vitiligo epidermis and released IFN-γ when exposed to external and internal stress. The released IFN-γ then stimulates CXCL10 production by keratinocytes, which induces both melanocyte apoptosis via CXCR3 and the migration of CD8 + cytotoxic T cells (CTL) to the skin^31^. Significant increases in CXCL10, which elicit very low levels of immune reaction, has been reported in non-depigmented and perilesional vitiligo skin^32^. We showed that CXCL10 did not affect GPNMB expression (Fig. S6a) indicating that the effect of IFN-γ is not mediated by CXCL10 induction, rather imposes a direct effect via IFNGR (Figs. 6 and S7).

There have also been many reports confirming elevated levels of circulating IL-17 and increased numbers of Th17 lymphocytes in patients with non-segmental vitiligo^33–35^. Furthermore, IL-17 has been reported to damage melanocytes^36^.

We were impressed, therefore, to find that both of vitiligo-associated IFN-γ and IL-17A downregulated keratinocyte-GPNMB. Melanocyte-GPNMB has been reported to play a role in cell adhesion with keratinocytes through integrin^1^. Since E-cadherin has been reported to be expressed in human melanocytes^27^, it is speculated that keratinocyte-GPNMB may also contribute to cell adhesion with melanocytes through this E-cadherin, provided the fact that over 90% of the total GPNMB in NHEKs was in the cell-associated form (Figs. 4 and S2). IFN-γ and IL-17A exerted no influence on the shedding of cell-associated GPNMB, whereas both decreased the GPNMB expression itself, possibly loosening the interaction between the keratinocytes and melanocytes to form the floating melanocytes in vitiligo. Integrin, CD44s and α1 subunit of Na + /K + ATPases are all candidate melanocyte GPNMB receptors, as all of them are expressed in melanocytes and potentially bind with GPNMB^20,21,37–40^. Note, however, that dysfunction of specific binding between melanocytes and keratinocytes, but not among keratinocytes, is a requisite explanation for the vitiligo pathology, given that depigmentation is the only observable abnormality of vitiligo skin.

We cannot exclude the possibility that unknown vitiligo-related factors accelerate GPNMB shedding to produce sGPNMB, thereby affecting the vitiligo pathophysiology by modulating the functions of keratinocytes and/or melanocytes. sGPNMB has been detected in melanocytes^19^, brain cells^20^, and cancer cells^29^ etc., and is released in the extracellular spaces and accordingly may play important roles in distant cells or tissues^19,22^. Alternatively, the disappearance of keratinocyte-GPNMB in vitiligo lesions might be induced via the loss of unknown soluble factors which are released from healthy melanocytes to maintain the keratinocyte-GPNMB.

GPNMB might also be involved in the melanocyte maintenance or survival against the oxidative stress because it has been reported that macrophage-derived GPNMB played an important role in dermal wound healing through mesenchymal stem cells (MSCs) in the skin^41,42^, and the extracellular fragment of GPNMB showed neuroprotective effects in neuronal cell line^20^. Both of these observed effects were activated via PI3K/Akt and MEK/ERK pathways, the former through CD44 and the latter through the α-subunit of Na^+^/K^+^-ATPase, as receptors.

The roles of GPNMB in vitiligo and in healthy skin will be better elucidated by further *in vivo* and *in vitro* investigations into the functions and regulatory roles of GPNMB in keratinocytes and melanocytes.

In conclusion, we have demonstrated that cultured NHEKs express GPNMB and release sGPNMB by shedding, possibly via ADAM10, and that the basal keratinocytes of healthy human skin express GPNMB. We have also demonstrated that the disappearance of keratinocyte-GPNMB in vitiligo lesions is characteristic of vitiligo depigmentation, because the GPNMB signals remained positive in the lesional epidermis of nevus depigmentosus skins. IFN-γ may play a regulatory role in the pathological downregulation of keratinocyte-GPNMB. Decreased expression of GPNMB in keratinocytes may affect the maintenance or survival of melanocytes under oxidative stress, although further studies are needed to clarify the issue. These findings indicate a new target for vitiligo treatment focusing on the novel role of IFN-γ and IL-17 in downregulating keratinocyte-GPNMB. Further investigations to clarify the functions and regulatory actions of GPNMB in keratinocytes, as well as melanocytes, will be needed to confirm the implications of this study.

## Materials and Methods

### Cell culture

Normal human epidermal keratinocytes (NHEKs) purchased from Kurabo Industries Ltd., Japan were cultured in a 75 cm^2^ flask using a defined media (HuMedia-KG2; Kurabo Industries Ltd., Japan) containing 10 µg/ml insulin, 0.1 ng/ml human epidermal growth factor (hEGF), 0.67 µg/ml hydrocortisone hemisuccinate, 50 µg/ml gentamicin, 50 ng/ml amphotericin, 0.4% bovine pituitary extract (BPE), and 0.06 mM calcium chloride, and maintained at 37 °C under a humidified atmosphere of 95% air and 5% CO_2_. Normal human epidermal melanocytes (NHEMs) purchased from Lonza, MD, USA were cultured in a melanocyte basal medium (MBM-4; Lonza, MD, USA) supplemented with the ingredients specified in the manufacturer’s instructions and maintained under the same appropriate atmospheric conditions described above. Melanoma cells (C32TG, G361, Mewo) kindly provided by Professor Dr. Higashiyama (Ehime University, JAPAN) were cultured in RPMI-1640 medium (FUJIFILM Wako Pure Chemical Co., Tokyo, JAPAN) together with 10% FBS (FUJIFILM Wako Pure Chemical Co., Tokyo, JAPAN) and 1% Penicillin-Streptomycin (Nakalai Tesque, Kyoto, JAPAN), and maintained under the same appropriate atmospheric conditions described above. The cells used for the experiments were cultured and maintained in collagen-coated 6-well plates (Corning Incorporated, NY, USA). NHEKs and NHEMs from the third to fifth passage were used for the experiments.

### Human skin specimens

Clinical procedures were performed in accordance with the guidelines of Helsinki Declaration. Human skin specimens were taken from the subjects who had given their written informed consent to participate in the study. The protocol was approved by the ethics committee of the Osaka University Faculty of Medicine in Japan (No. 10339). The information of skin donors was listed in a table (Supplementary Table 1) in Supplementary Information.

### Knockdown of GPNMB and STAT1 by transfecting with small interfering RNA (siRNA)

Three GPNMB siRNA sequences were examined for their effects on the downregulation of GPNMB expression, and all the three sequences were shown to have ability to interfere GPNMB mRNA (Supplementary Fig. S2). However, the siRNA no. 21 was used for the subsequent analysis because it was found to be the most effective. NHEKs and NHEMs were transfected with 5 nM GPNMB siRNA (No. 21) and a negative control siRNA (FlexiTube siRNA; Qiagen, CA, USA) using HiPerFect transfection reagent (Qiagen, CA, USA) according to the manufacturer’s instructions. The knockdown was verified by western blotting with an antibody specific for GPNMB. The siRNA sequences (No. 21) of GPNMB were as follows: 5′-GGAGCUGAGUAGGAUUCCUGAUGAA-3′ (forward) and 5′-UUCAUCAGGAAUCCUACUCAGCUCC-3′ (reverse). On the other hand, the STAT1 siRNA at 5 nM level and a negative control siRNA (FlexiTube siRNA; Qiagen, CA, USA) were transfected into NHEKs using HiPerFect transfection reagent (Qiagen, CA, USA) according to the manufacturer’s instructions.

### RNA isolation

Total RNA was extracted from NHEKs and NHEMs using an RNeasy Mini Kit (Qiagen, CA, USA) according to the manufacturer’s instructions. The quality and quantity of total RNA were determined by a NANODROP 2000c Spectrophotometer (Thermo Fisher Scientific, MA, USA).

### Real-time polymerase chain reaction (PCR)

Total RNA was reverse transcribed to complementary DNA (cDNA) using a PrimeScript RT reagent kit (Takara Bio, Otsu, Japan). Relative semi-quantitative real-time PCR was carried out in a Thermal Cycler Dice Real Time System TP800 (GE Healthcare, Buckinghamshire, UK) using the SYBR Premix Ex Taq II system (Takara Bio, Otsu, Japan) according to the manufacturer’s instructions. The thermal cycling conditions were as follows: 30 s at 95 °C, followed by 40 cycles of two-step PCR at 95 °C for 5 s and 60 °C for 30 s, followed by a single cycle of dissociation steps performed at 95 °C for 15 s, 60 °C for 30 s, and 95 °C for 15 s. Ribosomal protein S18 (RPS18) mRNA was used as control. The primer sequences were as follows: for GPNMB, 5′-TCCAGATGACAGACGTCCTGATG-3′ (forward) and 5′-TCTGGGTGATCTCGCAGGTG-3′ (reverse); and for RPS18, 5′-TTTGCGAGTACTCAACACCAACA-3′ (forward) and 5′-CCTCTTGGTGAGGTCAATGTCTG-3′ (reverse). The delta-delta-CT method was used to compare the differences of mRNA expressions among the different experimental groups.

### Cell immunostaining

NHEKs and NHEMs were seeded at 5 × 10^4^ cells/dish (35 mm dish), incubated for 72 hrs, and fixed with 4% paraformaldehyde for 10 min. After the cells were permeabilized with 0.2% triton for 5 min, they were blocked using a mixture containing 1% BSA and 20% heat-inactivated serum for 30 min. The cells were then incubated overnight at 4 °C with Human Osteoactivin/GPNMB Antibody (1:200, antigen affinity-purified polyclonal goat IgG, catalog number: AF2550; R&D Systems, MN, USA) as the primary antibody. Next, the cells were incubated with Alexa Fluor 546 Donkey Anti-Goat IgG (1:500, Thermo Fisher Scientific, MA, USA) as the secondary antibody at room temperature for 1 hr. After incubating the cells with Hoechst for 5 min, images were taken using a confocal microscope (Olympus, Tokyo, Japan).

### Immunohistochemistry

Skin samples were fixed in 10% formaldehyde for routine processing and paraffin embedding, then sectioned (4 µm) and subjected to immunofluorescence staining. Antigens were activated by a microwave treatment over 95 °C for 16 min in Tris-EDTA buffer (10 mM Tris, 1 mM EDTA, pH 9.0). Human Osteoactivin/GPNMB Antibody (catalog number: AF2550; R&D Systems, MN, USA) was used as a primary antibody, and Alexa Fluor 555 Rabbit-Anti Goat IgG was used as a secondary antibody. Monoclonal Mouse Anti-Human Melan A Antibody (Code: M7196; DAKO GmbH, Jena, Germany) was used for Melan-A staining. Nuclear staining was performed for 30 sec using WAKO Mayer’s Hematoxylin solution (Wako Pure Chemical Corporation, Osaka, Japan).

### Western blot analysis

NHEKs, NHEMs, and melanoma cells were lysed with a lysis buffer mix containing Passive Lysis Buffer (Promega Corporation, WI, USA), Protease Inhibitor Cocktail Tablets (Roche Diagnostics GmbH, Mannheim, Germany), Phosphatase Inhibitor Cocktail Tablets (Roche Diagnostics GmbH, Mannheim, Germany), and Phenylmethylsulfonyl Fluoride (PMSF; Sigma Aldrich, MO, USA). The total protein concentration was measured with a Bio-Rad Protein Assay Dye Reagent Concentrate Kit (Bio-Rad Laboratories, CA, USA) using different concentrations of Bovine Serum Albumin (BSA; Sigma Aldrich, MO, USA) as standards. The same concentrations of different protein samples (glycosylated or deglycosylated) were treated with a sample buffer containing 10% β-mercaptoethanol and incubated at 95 °C for 5 min. After subjecting the proteins to Sodium Dodecyl Sulfate Polyacrylamide Gel Electrophoresis (SDS-PAGE) in 4–20% gradient gels (Mini-PROTEAN TGX Gels; Bio-Rad Laboratories, CA, USA), the separated proteins were transferred onto Polyvinylidene Difluoride (PVDF) membrane (Trans-Blot Turbo Transfer Pack; Bio-Rad Laboratories, CA, USA).

After blocking with a blocking reagent (PVDF Blocking Reagent for Can Get Signal; Toyobo Co. Ltd., Osaka, Japan), the membrane was incubated with two primary antibodies: Human Osteoactivin/GPNMB Antibody (1:1000; antigen affinity-purified polyclonal goat IgG, catalog number: AF2550; R&D Systems, MN, USA) and mouse Monoclonal Anti-β-Actin antibody (1:1000; Sigma Aldrich, MO, USA). Next, the membrane was incubated with two secondary antibodies: Rabbit Anti-Goat IgG H&L (HRP) (1:10000; Abcam, USA) and Amersham ECL Anti-Mouse IgG, horseradish peroxidase linked species-specific whole antibody from sheep (1:10000; GE Healthcare, Buckinghamshire, UK). After the membrane was incubated with its substrate (Amersham ECL Prime Western Blotting Detection Reagent; GE Healthcare, Buckinghamshire, UK), the bands were visualized using an imaging system (Molecular Imager ChemiDoc XRS Plus; Bio-Rad Laboratories, CA, USA). For deglycosylation, the cell lysate proteins was treated with a glycosidase enzyme (PNGase F PRIME; N-Zyme Scientifics, PA, USA) and incubated at 37 °C for 3 hrs. For detection of phospho-STAT1 expression, Phospho-Stat1 (Ser727) (D3B7) Rabbit mAb (1:1000; Cell Signaling Technology, USA) was used as a primary antibody.

### Enzyme linked immunosorbent assay (ELISA)

NHEKs were cultured and maintained on 6-well plates and conditioned medium was collected from the cell culture. Cellular proteins were prepared from the corresponding wells by lysing the cells using a lysis buffer as described in the previous section. The absolute concentrations of the soluble and cell-associated GPNMBs were measured by ELISA (Osteoactivin Human ELISA Kit; Abcam, Cambridge, UK) according to the manufacturer’s instructions.

### Statistical analysis

Data were expressed as mean ± standard deviation (M ± SD) and analyzed by the Statistical Package for the Social Sciences (SPSS) software (IBM Corporation, NY, USA). Statistical comparisons were performed by the student’s t-test or one-way ANOVA followed by the Dunnett’s test or Tukey’s test. A value of p < 0.05 was considered statistically significant.

## Supplementary information

Supplementary Information.
