# Supplementary material for: GPNMB is expressed in human epidermal keratinocytes but disappears in the vitiligo lesional skin
Source: Sci Rep. 2020 Mar 18;10:4930. doi: 10.1038/s41598-020-61931-1 (PMC7080742; doi:10.1038/s41598-020-61931-1)
Supplement: Supplementary file 1 — Supplementary Information. [file 41598_2020_61931_MOESM1_ESM.pdf]

Supplementary information

GPNMB is expressed in human epidermal keratinocytes but disappears in the vitiligo lesional skin

Kazal Boron Biswas[1,2], Aya Takahashi[3], Yukiko Mizutani[1], Satoru Takayama[1,2], Asako Ishitsuka[1], Lingli Yang[3], Fei Yang[3], Arunasiri Iddamalgoda[1,2], Ichiro Katayama[3], and Shintaro Inoue[1]

[1]Department of Cosmetic Health Science, Gifu Pharmaceutical University, Gifu, Japan. [2]Department of Research and Development, Ichimaru Pharcos Co. Ltd., Motosu, Gifu, Japan. [3]Department of Dermatology, Osaka University School of Medicine, Osaka, Japan. Correspondence and requests for materials should be addressed to I.K. (email: katayama@derma.med.osaka-u.ac.jp), and S.I. (email: inoshin@gifu-pu.ac.jp)

Figure S1.

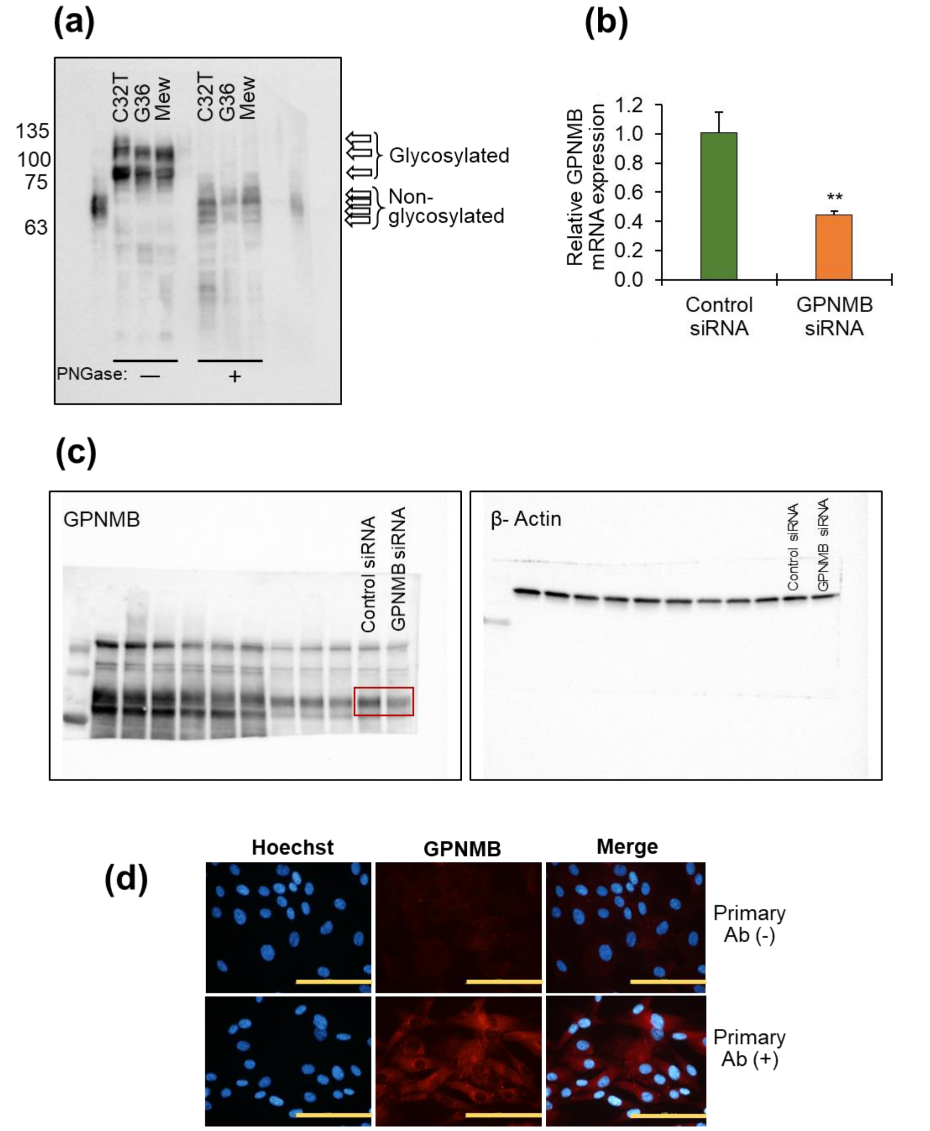

Fig. S1. Characterization of the specificity of anti-human GPNMB antibody. (**a**) Melanoma cells were cultured and then lysed with cell lysis buffer. Total protein was isolated from the cell lysate, and western blot analyses were carried out in the presence and absence of PNGase to identify the cell-associated GPNMB. (**b**) After transfecting normal human epidermal melanocytes (NHEMs) with siRNA for 48 hrs, the mRNA expression of GPNMB was measured by PCR. Data were expressed as mean±SD (n=3). ***P*<0.01 *vs* control (Student's t-test). (**c**) Under the same experimental conditions described in (b), total protein was isolated from the cell lysate and subjected to WB analysis in the presence of PNGase. (**d**) NHEMs were cultured on glass-bottom dishes and immunostained for GPNMB expression in the presence or absence of GPNMB primary antibody. The scale bar is equal to 100 μm.

Figure S2.

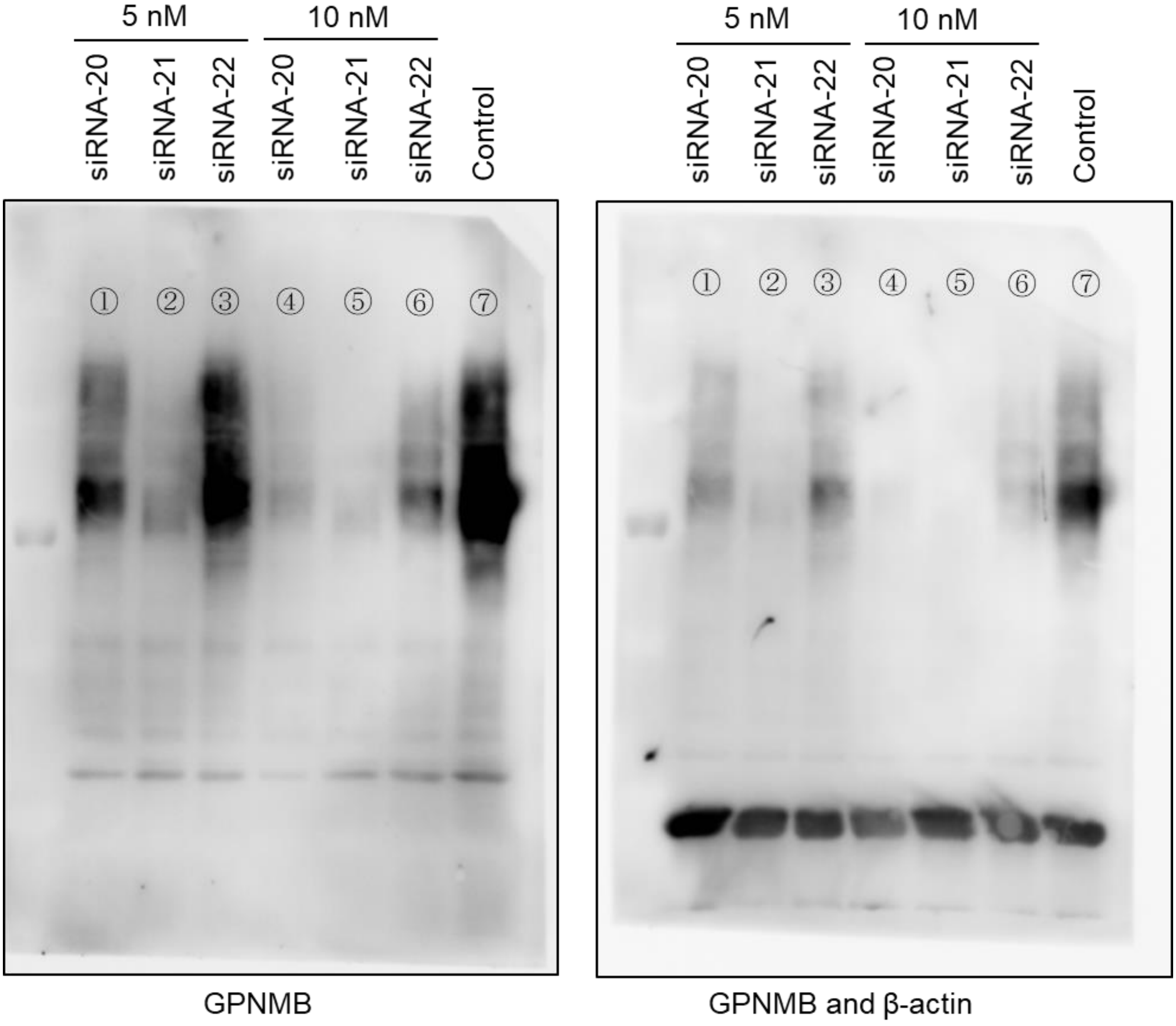

Fig. S2. Inhibition of GPNMB expression using different siRNAs in NHEKs. NHEKs were cultured in 24-well plates with the respective concentrations of GPNMB siRNAs for 3 days. Western blotting analysis was performed using antibodies specific to GPNMB and β-actin.

Figure S3.

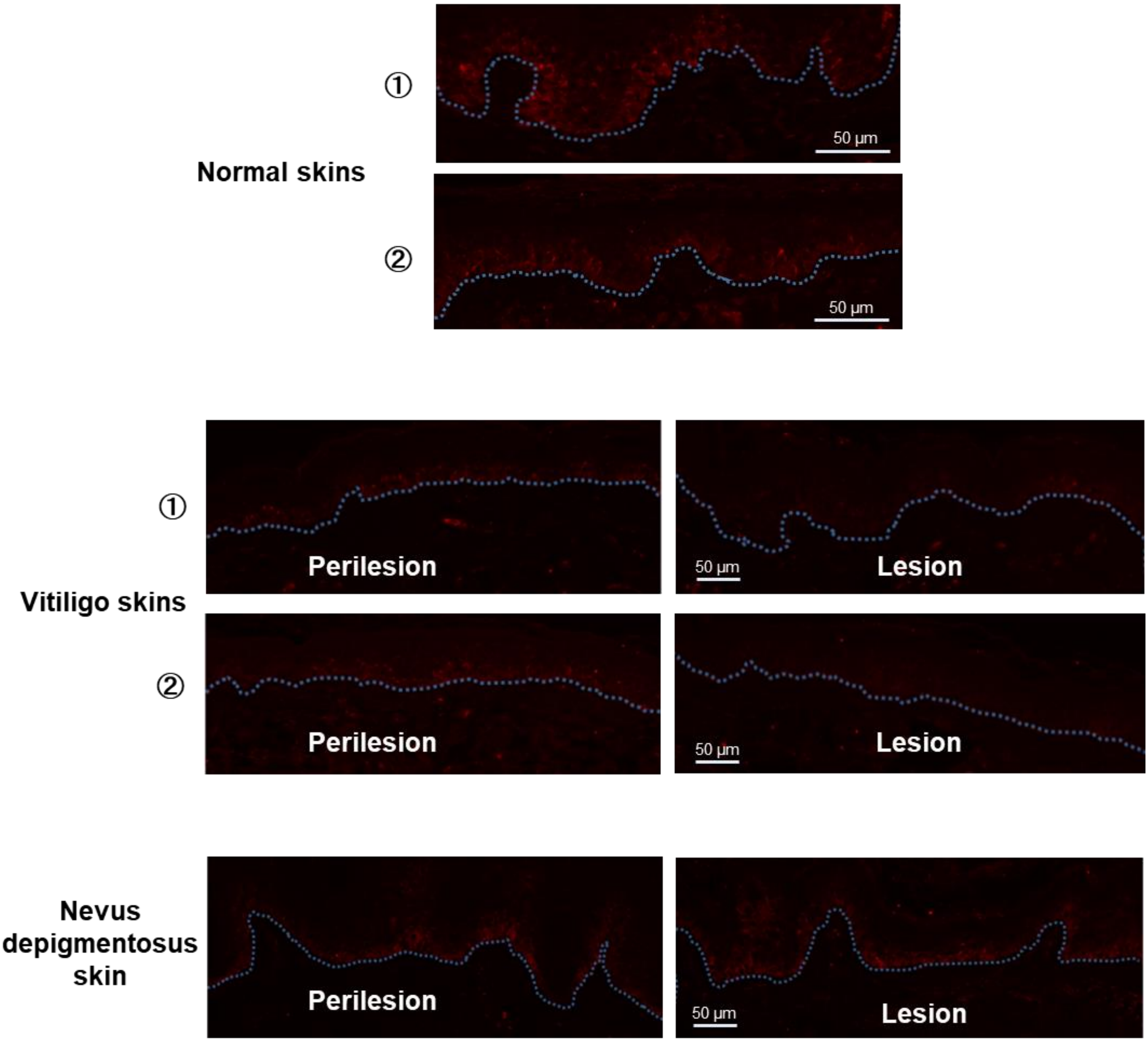

Fig. S3. Expression of GPNMB in different skin types. Skin samples collected from two normal human subjects (as controls), two vitiligo patients, and one nevous depigmentosus patient were immunostained using anti-human GPNMB antibody. The GPNMB was stained red.

Figure S4.

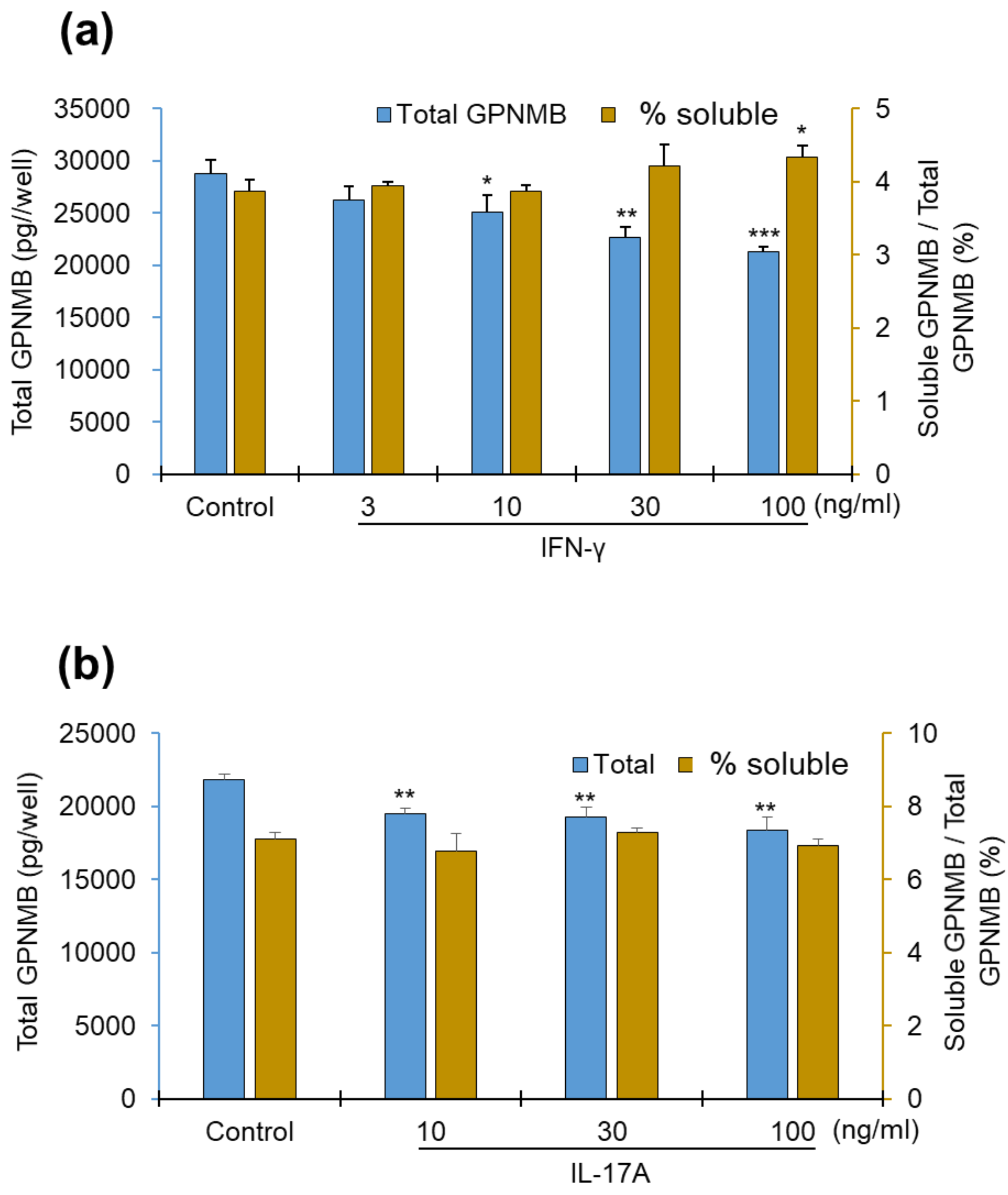

Fig. S4. Effects of IFN-γ and IL-17A on the expression and shedding of GPNMB in NHEKs. After 24 hrs of incubation with different concentrations of IFN-γ and IL-17A, soluble GPNMB from cultured medium and cell-associated GPNMB were measured by the ELISA method. Data were expressed as mean±SD (n=3). *P<0.05, **P<0.01, and ***P<0.001 vs control (one-way ANOVA followed by Dunnett's test).

Figure S5.

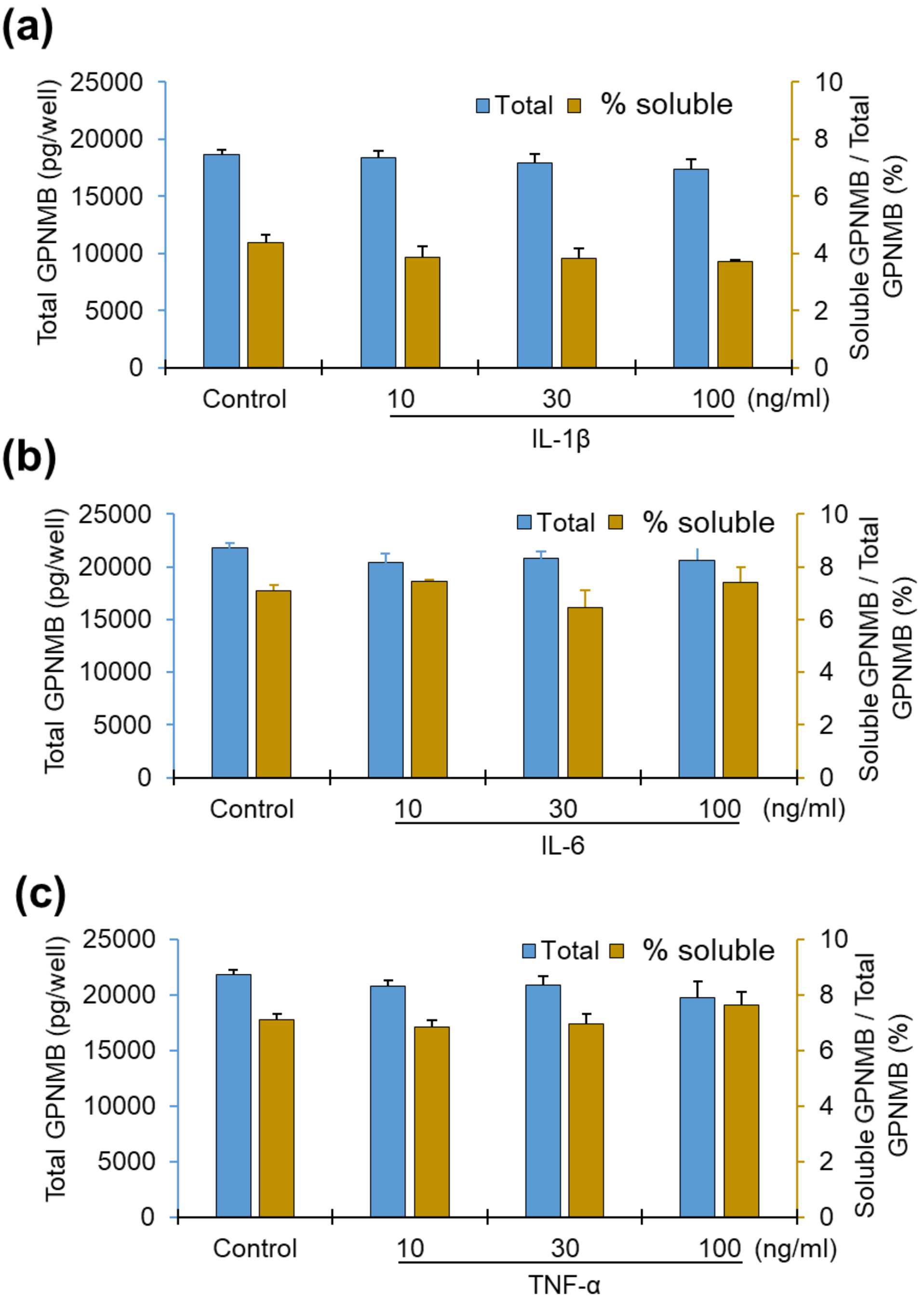

Fig. S5. Effects of IL-1β, IL-6, and TNF-α on the expression and secretion of GPNMB in NHEKs. NHEKs were cultured in the presence of different concentrations of IL-1β, IL-6, and TNF-α. After 24 hrs, soluble and cell-associated GPNMB were measured by ELISA. Data were expressed as mean±SD (n=3). Differences versus control were compared by performing one-way ANOVA followed by the Dunnett's test.

Figure S6.

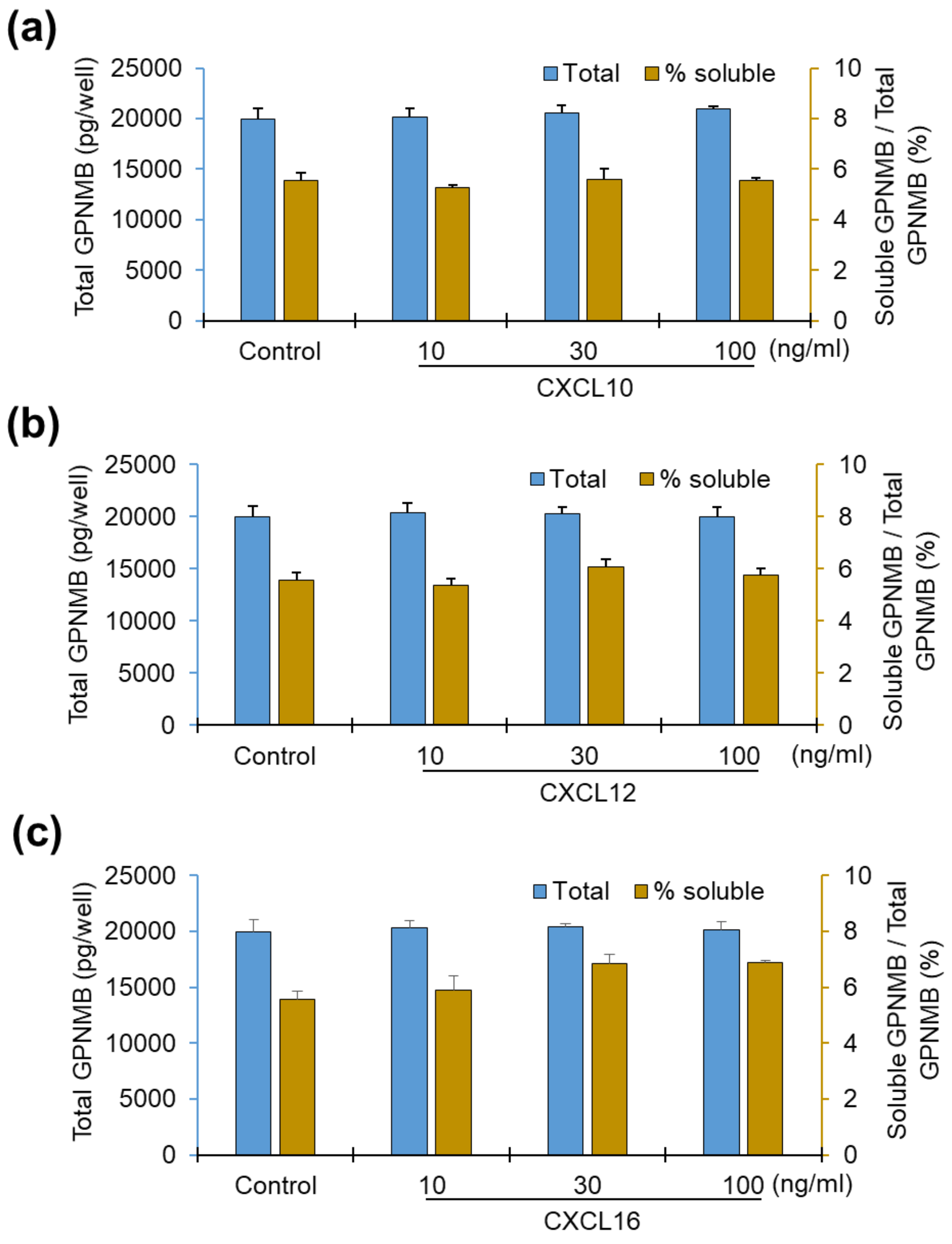

Fig. S6. Effects of CXCL10, CXCL12, and CXCL16 on the expression and release of GPNMB in NHEKs. NHEKs were cultured in the presence of different concentrations of CXCL10, CXCL12, and CXCL16. After 24 hrs, soluble and cell-associated GPNMB were measured by ELISA. Data were expressed as mean±SD (n=3). Differences versus control were compared by performing one-way ANOVA followed by the Dunnett's test.

Figure S7.

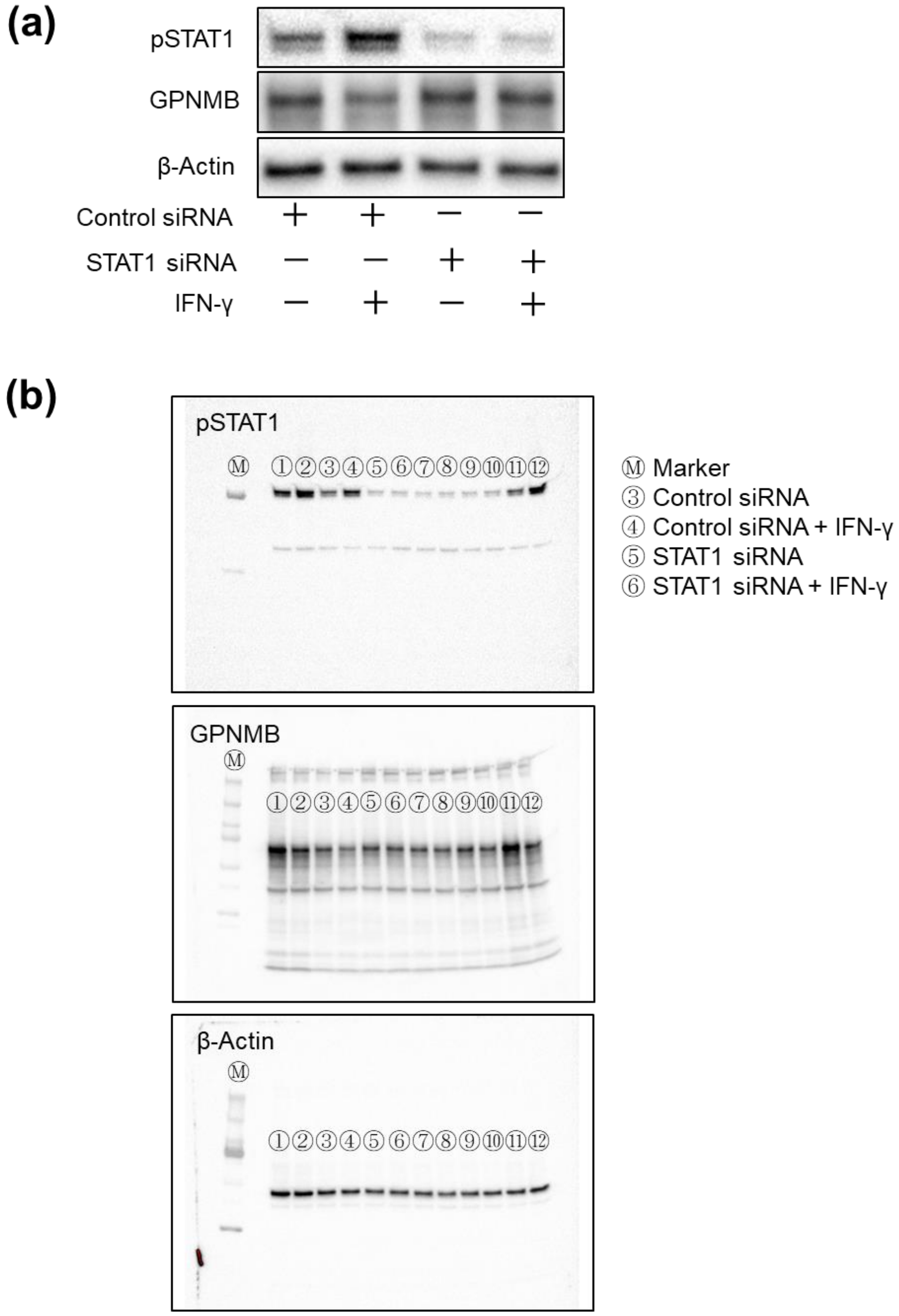

Fig. S7. Involvement of STAT1 in the IFN-γ-mediated inhibition of GPNMB expression in NHEKs. **(a)** STAT1 siRNA (5 nM) was transfected into NHEKs for 48 hrs followed by incubation with IFN-γ (30 ng/ml) for 24 hrs. Isolated protein was treated with PNGase for deglycosylation. Western blot analyses were done for the expression of pSTAT1 and GPNMB. **(b)** These figures represent the original and unprocessed full-length blots of Fig. S7 (a).

Figure S8.

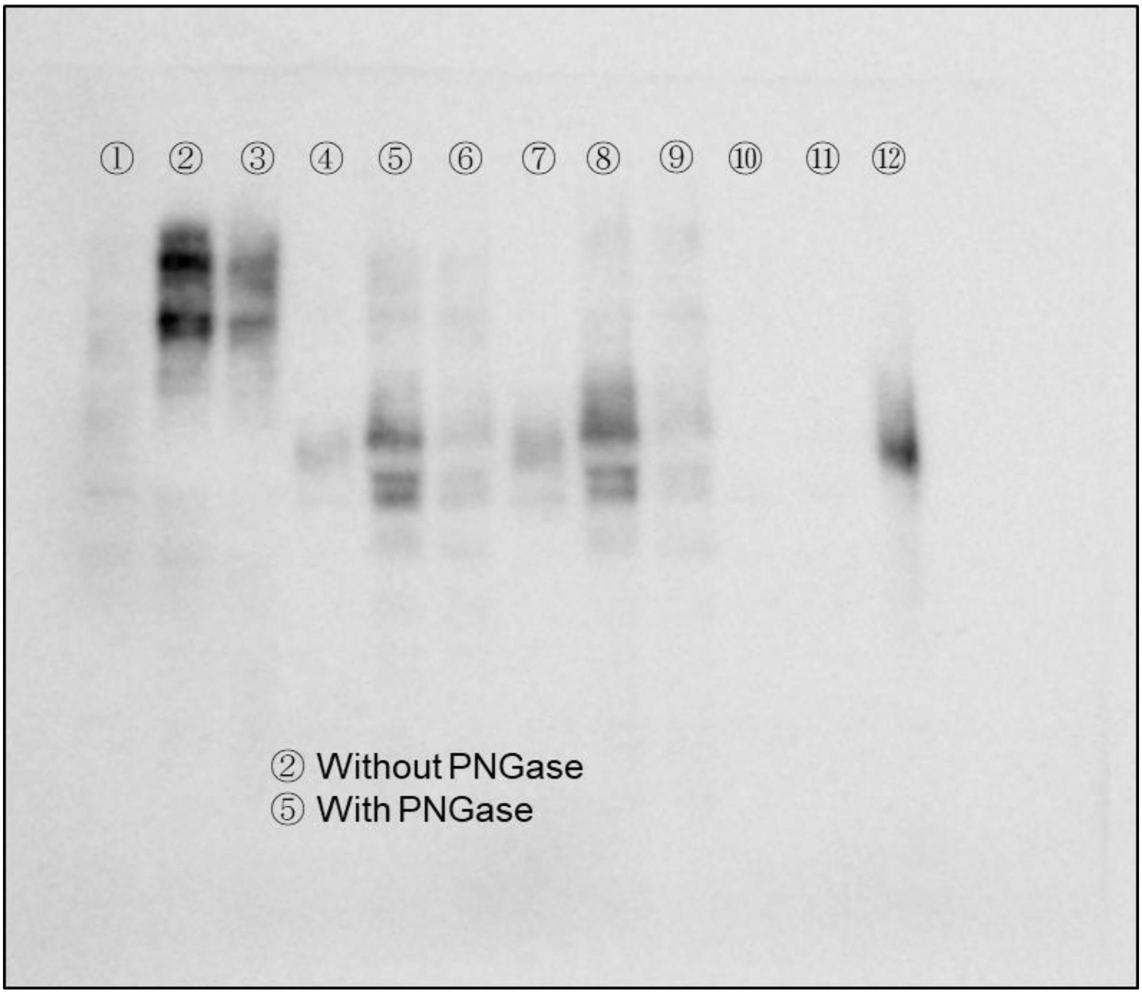

Fig. S8. This figure represents the original and unprocessed full-length blot of Figure 1a.

Figure S9.

a) GPNMB

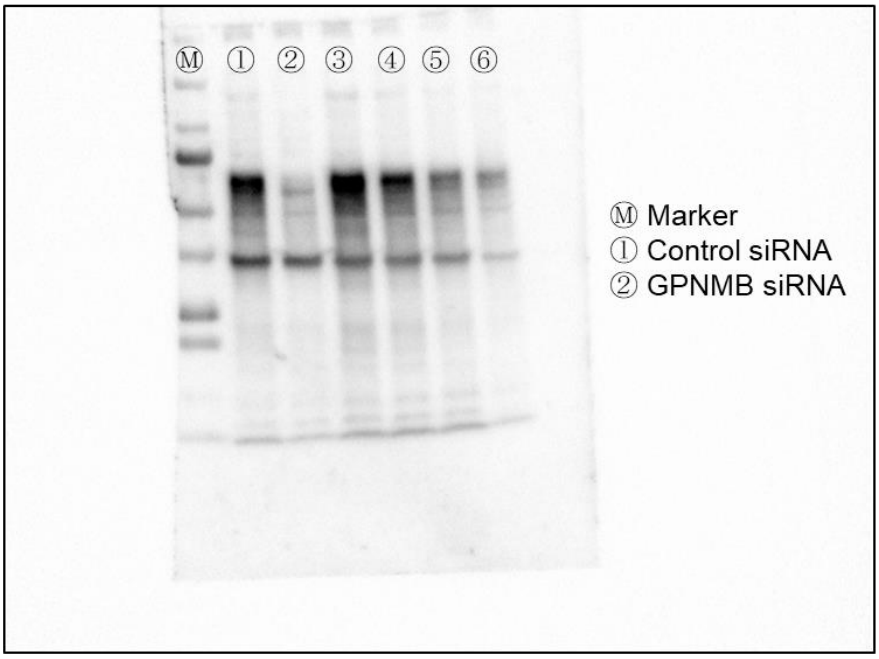

b) β-Actin

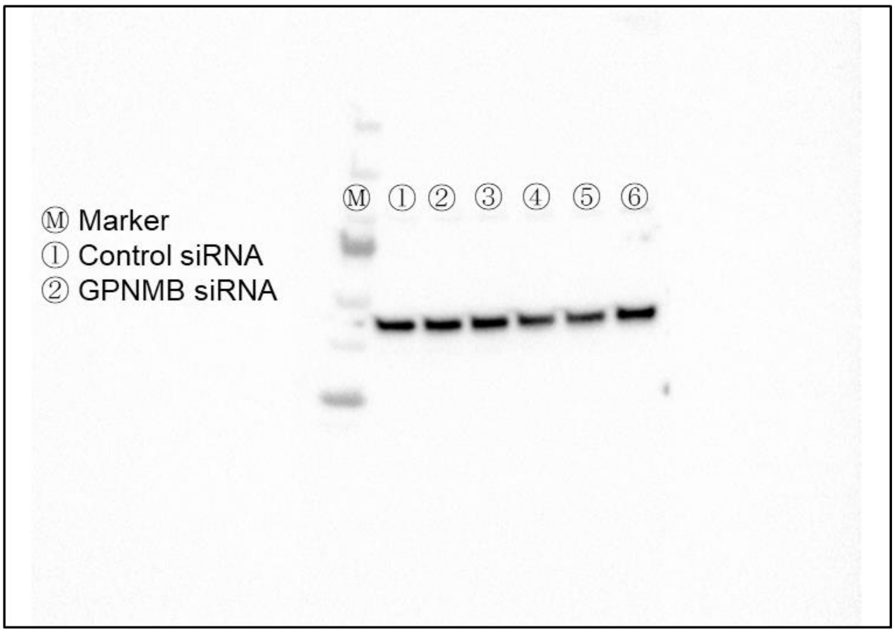

Fig. S9. These figures represent the original and unprocessed full-length blots of Figure 1c.

Figure S10.

a) GPNMB

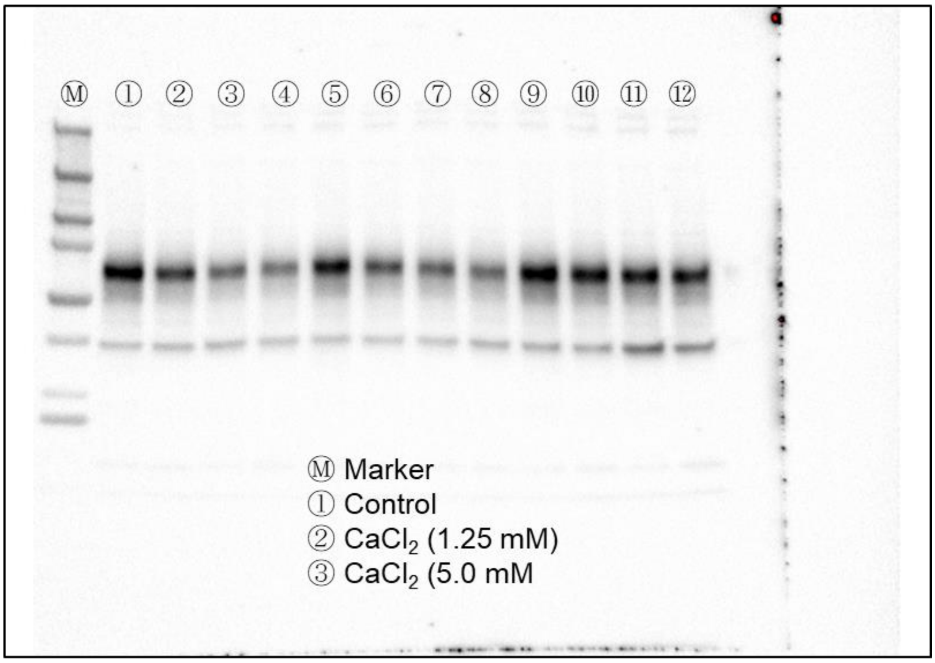

b) β-Actin

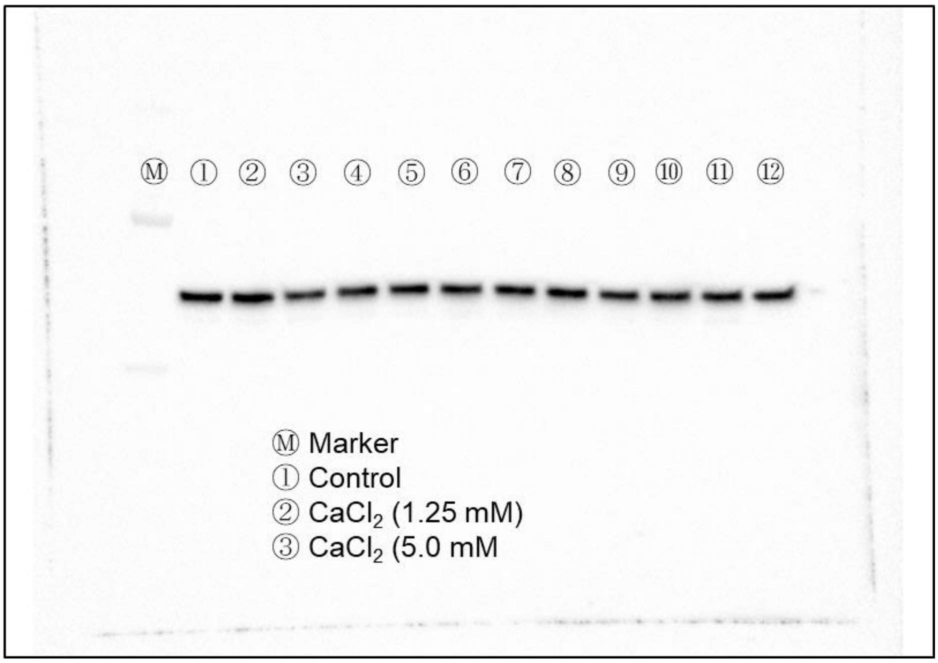

Fig. S10. These figures represent the original and unprocessed full-length blots of Figure 2b.

Figure S11.

a) GPNMB

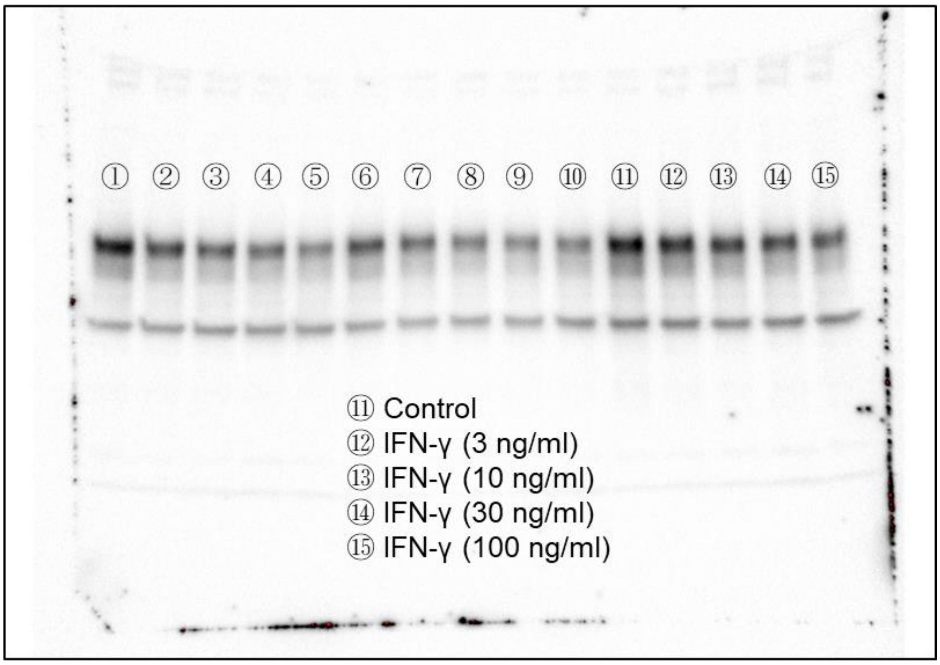

b) β-Actin

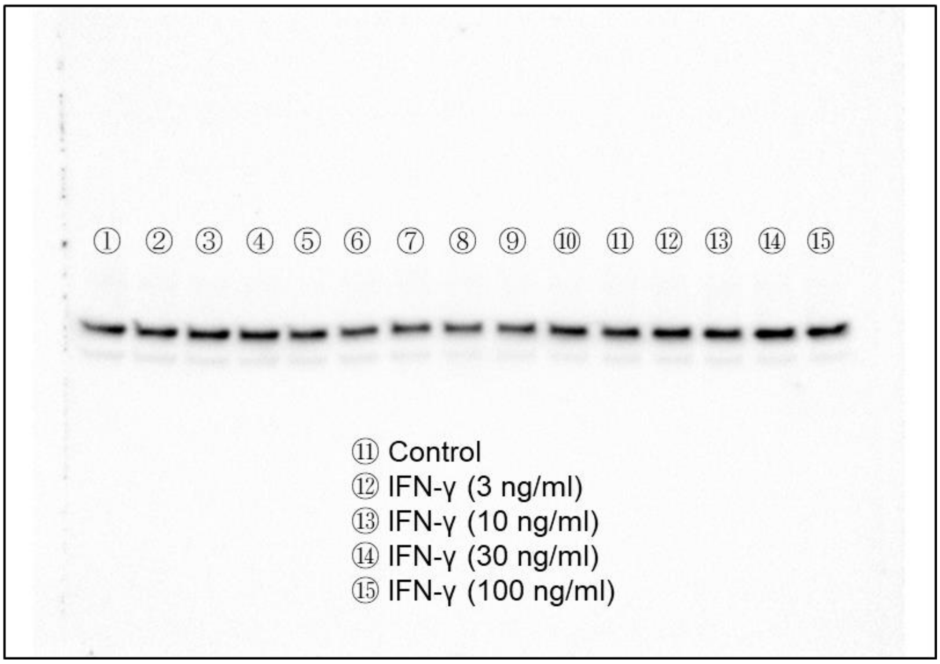

Fig. S11. These figures represent the original and unprocessed full-length blots of Figure 5b.

Figure S12.

a) GPNMB

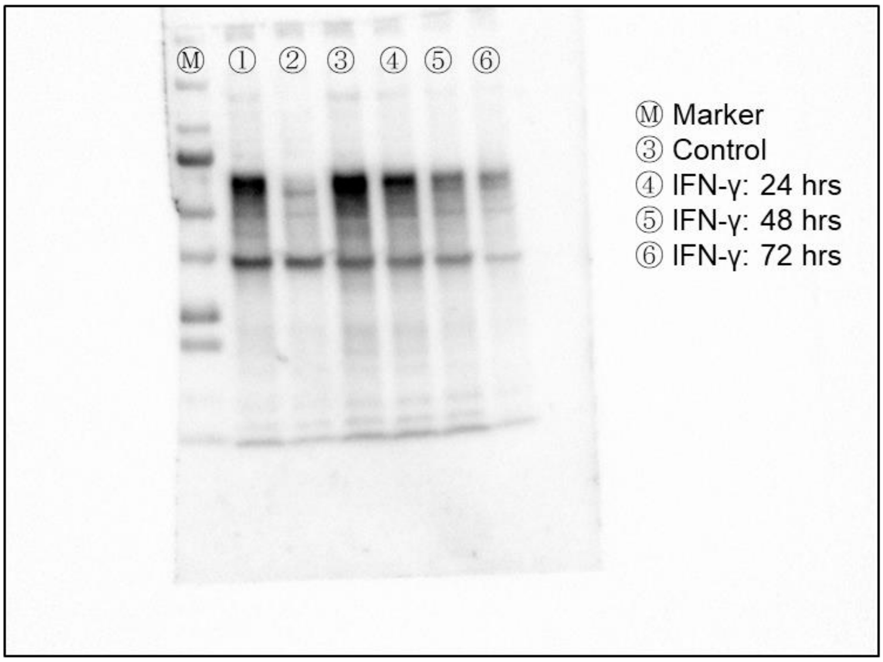

b) β-Actin

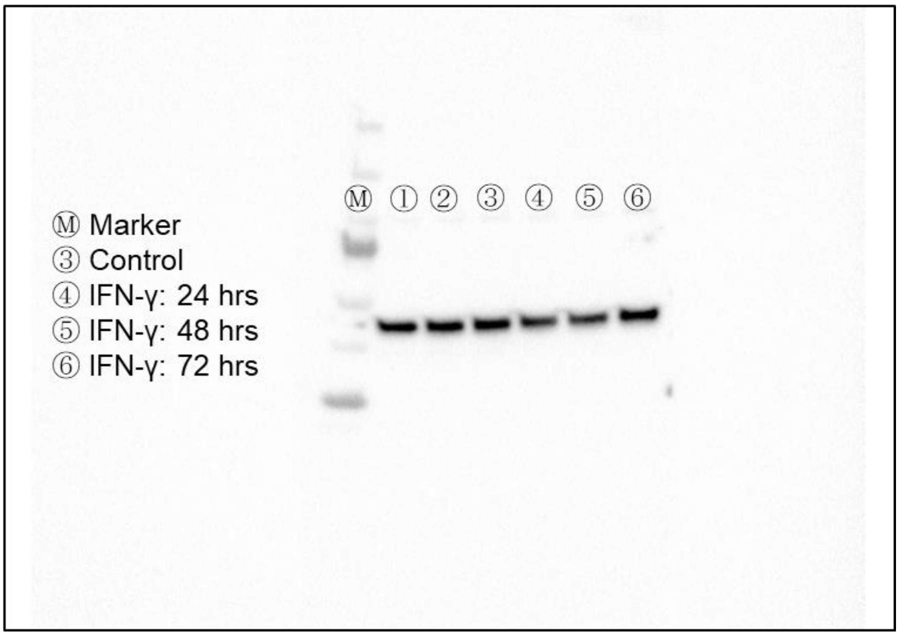

Fig. S12. These figures represent the original and unprocessed full-length blots of Figure 5d.

Figure S13.

a) GPNMB

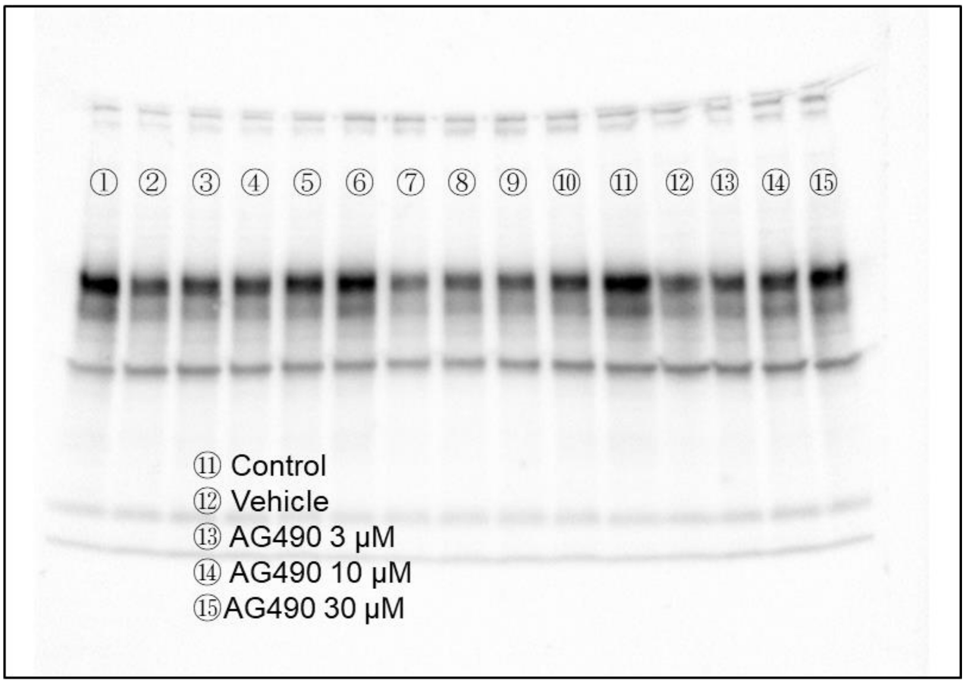

b) β- Actin

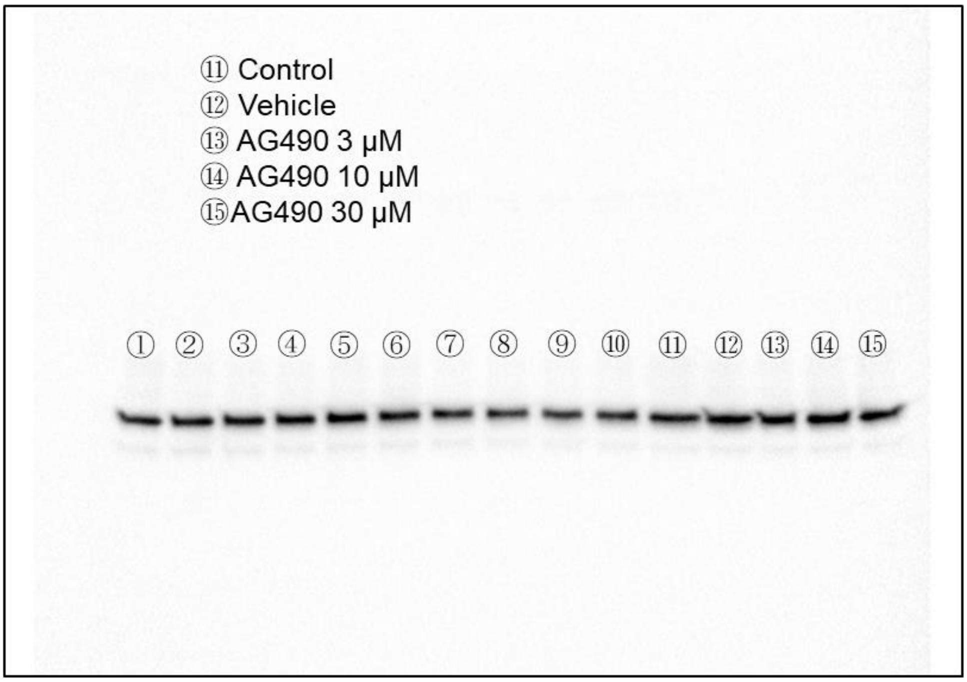

Fig. S13. These figures represent the original and unprocessed full-length blots of Figure 6b.

Supplementary Table 1. Information of skin donors

Figure No.	Age	Gender	Region
Fig 3a: Normal	70	M	Dorsal
Fig 3b, 3c: Vitiligo	49	F	Dorsum manus
Fig S3: Normal ①	48	M	Inguinal
Fig S3: Normal ②	59	F	Forehead
Fig S3: Vitiligo ①	47	F	Abdominal
Fig S3: Vitiligo ②	60	F	Mandibular
Fig S3: Nevus depigmentosus	3	M	Forearm
